# Co-Producing a Survey on Prospective Acceptability of Neuromodulation for Mental Health Conditions with Lived Experience Experts

**DOI:** 10.1192/bjo.2025.10239

**Published:** 2025-06-20

**Authors:** Sue Fen Tan, NIHR MindTech HealthTech Research Centre, Paul Briley

**Affiliations:** 1 Nottinghamshire Healthcare NHS Foundation Trust, Nottingham, United Kingdom; 2 University of Nottingham, Nottingham, United Kingdom

## Abstract

**Aims:** Non-invasive brain stimulation (“neuromodulation”) techniques, including transcranial magnetic stimulation (TMS) and transcranial electrical stimulation (TES), are used to modulate brain excitability and connectivity. TMS is approved for treating depression in the United Kingdom and preliminary evidence suggests that combining TMS and TES may enhance therapeutic effects. While neuromodulation is generally well-tolerated in research settings, its acceptability among the broader patient population remains unclear due to limited exposure, awareness, and information accessibility. Understanding prospective acceptability, defined as the perceived appropriateness of an intervention before its application, is crucial for improving treatment uptake and addressing concerns about safety and feasibility. We aimed to co-produce a survey with lived experience experts to assess the acceptability of individual and combined neuromodulation techniques among potential service users.

**Methods:** The study was co-developed with our Neuromodulation Experts-by-experience Advisory patient and public involvement (PPI) group. We underwent three rounds of iterative feedback to refine the survey focus, structure, and questions. A scoping review of existing literature on prospective acceptability of neuromodulation techniques informed the content, alongside the Theoretical Framework of Acceptability. Given the novelty of combined (TMS+TES) neuromodulation, no prior informational materials exist. PPI members advised it was critical to produce accompanying videos and leaflets to briefly illustrate the different neuromodulation techniques. The video scripts and leaflet content were produced in collaboration with three PPI members who tried the neuromodulation techniques, to avoid rehearsed scripts and ensure honest reviews of the techniques.

**Results:** The final survey version was adapted to maximise clarity of questions, engagement, and completion rates. The survey incorporated questions on awareness, perceived effectiveness, ethical considerations, and practical burden of different neuromodulation techniques. Online and paper versions of the survey were created to ensure accessibility. We successfully produced three information videos within 90-second target duration featuring PPI members and lead researchers. We developed a supplementary infographic leaflet for enhanced comprehension and accessibility.

**Conclusion:** Engaging stakeholders through PPI was instrumental in developing the survey to ensure accessibility and relevance for diverse participants with lived experience of mental health conditions. End-user involvement in the design process improved survey comprehensibility, highlighting the importance of co-production in developing effective research tools. Findings from this survey will provide insights into the acceptability of novel neuromodulation techniques, ultimately informing future clinical implementation and patient-centred research strategies.

